# Evaluation of Random Forests (RF) for Regional and Local-Scale Wheat Yield Prediction in Southeast Australia

**DOI:** 10.3390/s22030717

**Published:** 2022-01-18

**Authors:** Alexis Pang, Melissa W L Chang, Yang Chen

**Affiliations:** 1 School of Agriculture and Food, Faculty of Veterinary and Agricultural Sciences, The University of Melbourne, Parkville 3010, Australia; Melissa_CHANG@sfa.gov.sg (M.W.L.C.); y.chen@csiro.au (Y.C.); 2 Singapore Food Agency, JEM Office Tower, 52 Jurong Gateway Road, #14-01, Singapore 608550, Singapore; 3 CSIRO Data61, Goods Shed North, 34 Village St., Docklands 3008, Australia

**Keywords:** wheat, yield prediction, random forests, satellite imagery, Normalized Difference Vegetation Index (NDVI)

## Abstract

Wheat accounts for more than 50% of Australia’s total grain production. The capability to generate accurate in-season yield predictions is important across all components of the agricultural value chain. The literature on wheat yield prediction has motivated the need for more novel works evaluating machine learning techniques such as random forests (RF) at multiple scales. This research applied a Random Forest Regression (RFR) technique to build regional and local-scale yield prediction models at the pixel level for three southeast Australian wheat-growing paddocks, each located in Victoria (VIC), New South Wales (NSW) and South Australia (SA) using 2018 yield maps from data supplied by collaborating farmers. Time-series Normalized Difference Vegetation Index (NDVI) data derived from Planet’s high spatio-temporal resolution imagery, meteorological variables and yield data were used to train, test and validate the models at pixel level using Python libraries for (a) regional-scale three-paddock composite and (b) individual paddocks. The composite region-wide RF model prediction for the three paddocks performed well (*R*^2^ = 0.86, *RMSE* = 0.18 t ha^−1^). RF models for individual paddocks in VIC (*R*^2^ = 0.89, *RMSE* = 0.15 t ha^−1^) and NSW (*R*^2^ = 0.87, *RMSE* = 0.07 t ha^−1^) performed well, but moderate performance was seen for SA (*R*^2^ = 0.45, *RMSE* = 0.25 t ha^−1^). Generally, high values were underpredicted and low values overpredicted. This study demonstrated the feasibility of applying RF modeling on satellite imagery and yielded ‘big data’ for regional as well as local-scale yield prediction.

## 1. Introduction

Wheat is a key component of the Australian grain industry. Regional and national- scale wheat yield forecasting and prediction provide essential information to all parts of the value chain from farm production, aggregation, processing, distribution and through to the commodity markets, as well as governmental agricultural and economic departments. At the farm scale, this is the ability to monitor and predict crop health and, by extension, yields, in a spatially-variable manner within a farm paddock using NDVI facilitates precision variable-rate nitrogen application to achieve high production efficiencies and profitability [1]. The mainland southeast Australian wheat belt accounts for 53% of all wheat production regions [2], but is particularly vulnerable to significant volatility in yields due to climactic variability [3,4]. Therefore, this is a region that would benefit greatly from accurate yield prediction. Comprehensive and up-to-date reviews of crop yield prediction methods have been reported by [5,6].

High and ultra-high-resolution imagery using aerial platforms such as UAVs and manned aircraft can now provide high-precision quantitative information for crop monitoring of crop health and stresses at the sub-meter scale [7]. However, these techniques tend to be beyond the capabilities of normal producers or regional assessors and can also be limited by the spatial coverage and revisitation frequency (cadence) meaning that satellite-based data remain a critical component of regional and local-scale yield predictions. Cloud cover is a persistent problem [8] but this can be largely addressed with high-cadence imagery. Planet’s (www.planet.com; last accessed 17 July 2019) [9] constellation of Dove satellites offers an unprecedented observing potential of daily land surface imagery increasing the chances of acquiring cloud-free images for analysis, with an orthorectified spatial resolution of 3 m, enabling the detection of reflectance variations over very small distances and matching them with yield data [10]. This allows investigation of within-field yield variation which aids farmers in precision agriculture decisions. While somewhat limited in spectral resolution and range, PlanetScope imagery can bridge the spatio-temporal and spectral characteristics of MODIS (36 bands; 250 to 1000 m spatial resolution; daily revisit), Landsat 8 (9 bands; 30 m spatial resolution with 15 m for Band 8; 16-day revisit) and Sentinel 2 MSI (10 to 60 m spatial resolution; 5-day effective revisit) platforms that have recent multisensory data fusion strategies [11,12,13].

Machine Learning (ML)-driven approaches show much potential for the retrieval of key parameters such as biomass and soil moisture from satellite imagery [14]. While much previous work has focused on using Artificial Neural Networks (ANNs); the potential of random forests (RF) [15], being quicker and requiring fewer training dataset volumes, have yet to be comprehensively evaluated [14], particularly for dynamic, in-season wheat yield prediction at multiple scales. RF is a supervised ML algorithm based on decision- tree procedures to predict output classes based on patterns learnt in the training datasets. These involve building tree ensembles whose growth are controlled by randomized selection of (input-output) vectors from the training dataset; which are then assembled as classification or regression models to predict the most likely output class (or values) from the inputs of the test dataset with good accuracy and robustness to outliers with lower likelihood of generalization errors [15]. RF have the potential to generate better models compared to single decision-tree models [16], are more efficient computationally and therefore suitable for regional and global applications in agriculture [17] where Big Data dominates [18,19]. For instance, RF-driven yield prediction for sugar cane in Australia has been found to be more accurate and reliable than traditional approaches such as multiple linear regression [20,21]. For wheat yield prediction, methods ranging from a traditional crop-weather analysis model relating crop yield to stress (water, temperature) indices [22], to computationally-driven crop model simulation tools such as DSSAT and APSIM [23,24,25] have been used to varying degrees of success but require substantial calibration to reduce uncertainty. Recently, Feng et al. [26] adopted a hybrid approach combining a biophysical model and RF to improve dynamic yield forecasts for 29 sites across the New South Wales wheat belt and achieved good yield forecasting results (r = 0.87, *RMSE* = 0.64 t ha^−1^) based on the end of milk development stage. However, this study used NDVI derived from MODIS/MOD09GA surface reflectance composites at 500 m spatial resolution, precluding the assessment of intra-paddock variability.

Recent examples of RF-driven yield prediction include evaluating the effective use of RF at the global and country (USA) scale using wheat, maize and potato yield, climate, soil and fertilizer management datasets [27]; wheat biomass estimation in Jiangsu province of southern China using experimental plots and vegetation indices (VIs) from 30 m resolution multispectral imagery from HJ-1A/B satellites [28]; broad-scale wheat yield prediction over nine agricultural divisions in north China using Terra MODIS MOD13Q1 data, where RF was found to be one of the top best-performing ML algorithms [29]. These studies demonstrated the higher performance, robustness and accuracy of RF compared to statistical models, artificial neural networks (ANNs) and support vector regressions (SVRs). Furthermore, work on the use of ML techniques for within-farm wheat yield forecasting has been found to be still in their early stages [30,31] and therefore can provide novel and accurate information to aid farmers’ precision agriculture decision-making such as variable-rate nitrogen or phosphorus application for improved production efficiency and sustainability [32,33,34] as well as downstream stakeholders in the grain industry.

The main objective of this research was to evaluate the integration of ML (RFR) algorithms, high-resolution satellite imagery with multiple field and weather data to develop advanced, data-driven yet generalizable models for wheat yield prediction for wheat-growing paddocks in different parts of southeast Australia. This would therefore develop a foundation for developing region-centric algorithms for national-scale yield prediction. A key enabling objective was to build a parsimonious model (i.e., having a maximum predictive power using a minimum number of parameters) to predict yield in-season prior to, and up to harvest at various phenological stages while minimizing costs and complexity, and maximizing applicability to potential users (e.g., growers and agronomists).

## 2. Materials and Methods

**Figure 1:**
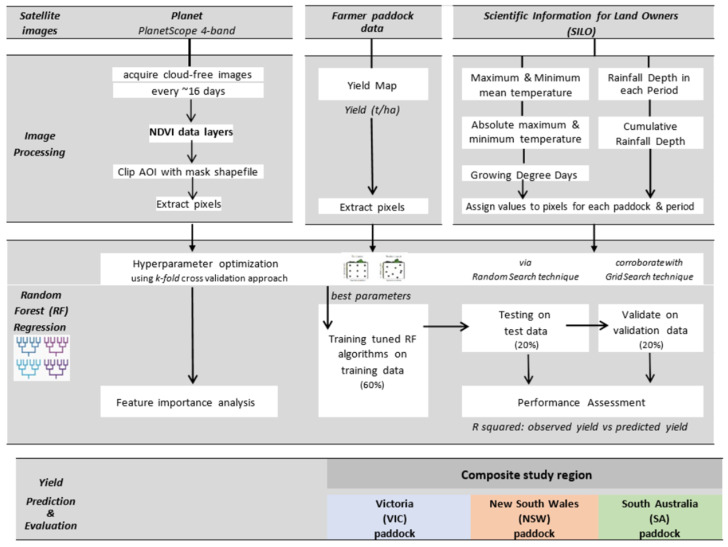
Summary of workflow processes and datasets used for building, testing and evaluating RF model wheat yield prediction method.

The project process workflow is summarized in Figure 1 and elaborated in the following sections.

### 2.1. Study Region Paddocks

**Figure 2:**
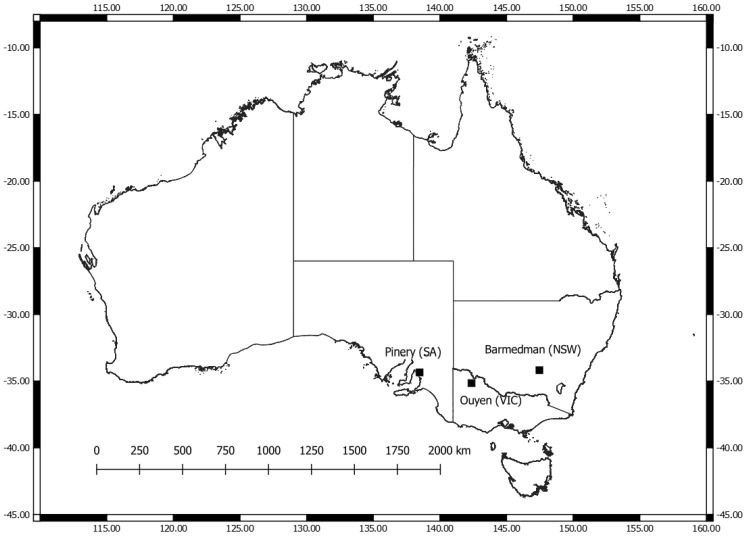
Location of study paddocks in southeast Australia, covering the states of Victoria (VIC), New South Wales (NSW) and South Australia (SA).

Spatially-distributed and referenced wheat yield values (t ha^−1^) were the pixel-level target variable for the RF prediction model. Three paddocks in southeast Australia viz. the states of Victoria (VIC), New South Wales (NSW) and South Australia (SA), that grew wheat in 2018 (Figure 2), were selected from a pool of private yield data collected from collaborating farmers; 5 m grid resolution yield maps were generated using a semi-automated procedure involving block kriging of yield monitor data, detailed in [35]. The verified yield maps were resampled to 3 m resolution to match with the PlanetScope imagery detailed below. The paddocks varied in hydroclimatic conditions, and soil characteristics and the preceding 3 years’ cropping/fallow sequences were likely to have affected fertility, water availability and crop residue cover leading into the 2018 season [36]. Different wheat varieties were also grown, adding another layer of complexity with which to test the robustness of the present technique. For instance, Kord is a mid-maturing variety that is robust to drought stresses, though not necessarily with the highest potential yields. Lancer is a mid to late-maturing variety suitable for early sowing with good resistance to lodging. Scepter is an early-mid season maturing type that has moderate resistance to lodging and one of the highest average yields of up to 3.0 t ha^−1^ in the SA wheat National Variety Trials (NVTs). These yield maps were used as training, testing and validation datasets for the RF model development [37].

According to study [38], 2018 was a particularly difficult growing season for southeast Australia cropping with the region experiencing rainfall in decile one range and temperatures in decile ten.

### 2.2. Planet^TM^ Satellite Imagery

NDVI data was used as one of the predictor variables (features); 16-day Periods spanning sowing to harvest dates for all three paddocks were created to constrain the temporal variability of the wide range of data and imagery, and also enable foreseen later work to compare with LANDSAT-based studies and imagery [39,40] (Table 1). In total, 41 PlanetScope Analytic Ortho Scene (Level 3B), cloud-free BGRN imagery (VIC: 13, NSW: 15, SA: 13) for the target paddocks were selected from available datasets, spanning the southeast Australia winter wheat-growing season, ~April to December 2018, from sowing to harvest. Ground Sample Distance (GSD) was 3.7 m and pixel dimensions were 3 m × 3 m. This spatial resolution was relevant to practical precision agronomic management by farmers (e.g., variable-rate fertilization), and harvesting header swath width varying between approx. 5 to 12 m. Normalized Difference Vegetation Index (NDVI) layers were generated for each scene using the Red (R) and Near Infra-Red (NIR) bands following [41]; see also [42,43] in QGIS 3.4 [44], before cropping to paddock boundaries.

In total, there were 377,475 pixels (3 m resolution; total area: 400 ha) across the VIC (188,865 pixels; 170 ha), NSW (67,830 pixels; 61 ha) and SA (120,780 pixels; 109 ha) paddocks. Areas covered by pixels analyzed were lower than actual paddock areas (Table 1) because the data were cropped internally from paddock boundaries to mitigate edge effects. 

The main dataset comprising all three paddocks was split into individual paddock datasets, giving two levels: regional-scale (three-paddock composite) and local-scale (individual paddock). All datasets were randomly divided into 60% training, 20% testing and 20% validation.

### 2.3. Weather Data

Location-specific daily weather data were compiled for each paddock from 5 km grid resolution values interpolated from local and regional networks of the Bureau of Meteorology and affiliated contractors’ weather station measurements, extracted from the Scientific Information for Land Owners (SILO) database (http://www.longpaddock.qld.gov.au/silo, last accessed 20 June 2019) [45], and assembled into the individual Periods (Table 2). For each Period, mean maximum and minimum, absolute maximum and minimum temperatures were prepared as predictor variables (features) that would help indicate heat or frost occurrence that could impact yield negatively; particularly pertinent at critical growth stages such as anthesis [46]. Growing degree days (GDD) corresponding to the imagery dates were also calculated and included as predictor variable [47]. Two rainfall datasets were prepared: rainfall depth (mm) in the preceding Period and cumulative rainfall depth (mm) since sowing date. Because of the coarse spatial resolution of the weather data, they were applied uniformly at the paddock scale for each Period by assigning the same value for all individual pixels within each paddock.

### 2.4. RF Model Development

Pandas software library functions for Python [48] were used for data preparation, manipulation and analysis. Time-series NDVI and weather data were used together as predictor variables. The NDVI data layers were parsed into CSV format with each cell value representing an individual pixel value. Weather variables were assembled as individual pixel values homogenous for each Period. Yield data (t ha^−1^) for individual spatially-referenced pixels were used as the target values for the prediction algorithms. All input and target values were indexed to retain their individual geographic locations to enable their reassembly for examination of their spatial distributions. 

The RF approach is an ensemble learning technique that makes predictions by combining decisions from a sequence of base models, with individual base models known as trees [49]. Hyper-parameters (e.g., weather and NDVIs) are tuned using the best cross-validation (CV) results. Random Forest Regression (RFR) was performed using the Scikit-learn machine learning module for Python [50]. Each tree in the RFR was built by using randomly selected variable sets from the training dataset with the final prediction for the testing datasets derived by averaging the tree outputs. Cross-validation was conducted to check the accuracy of the model on the independent validation dataset [51].

Calibration of each RFR model was done by hyperparameter tuning to obtain the optimal combination of: (i) number of trees in ensemble (*n_estimators*); (ii) maximum number of levels in each decision tree; (iii) maximum number of features considered for splitting a node, and (iv) method for sampling data points (with or without replacement). Random Grid Search was to incorporate a wide range of possible values and hyperparameter combinations in an unbiased manner, with superior computation times [52], an important consideration for mining large volumes of agricultural data. Twenty iterations of five-fold cross validation, with different model settings each time, were performed to facilitate model optimization and generalizability, while avoiding overfitting on the test dataset [50,53]. 

### 2.5. Feature Importance Analysis

Identifying and ranking the importance of individual features used in the RFR models we built, was conducted via Scikit-learn toolkit RF feature importance function, in order to understand the underlying dynamics contributing to model accuracy in yield prediction and ascertain their generalizability and meaningfulness [15,54,55]. To improve model performance while reducing the risk of overfitting, a forward-selection process was implemented following [21,56]. The optimum parameter combination giving the highest mean validation score was selected for model training. There was a need to balance performance against computational costs, even though model accuracy would expectedly increase with number of trees. To quantify and evaluate the tradeoffs made with different hyperparameter combinations, mean validation score was compared against number of trees, with the latter changed one at a time. Grid Search was then used for the selected numbers of trees to corroborate the optimality of the tuned settings, thus giving converged parameter settings of practical value.

## 3. Results

### 3.1. Regional (Composite) Yield Prediction

The RFR model developed for predicting yield of the three paddocks combined, i.e., at the regional scale, performed well with good generalizability across the VIC, NSW and SA locations. Table 3 compares the descriptive statistics of the observed and predicted yield datasets; the independent validation dataset. They were very similar, albeit with predicted minimum yield slightly higher, and maximum yield, slightly lower than the observed yield. Performance metrics shown in Table 4 demonstrate the good accuracy of the developed model. Notably, the adjusted *R*^2^ value and validated regression metric scores were similar, indicating good model generalization ability and absence of overfitting, performing well on unseen data. 

**Figure 3:**
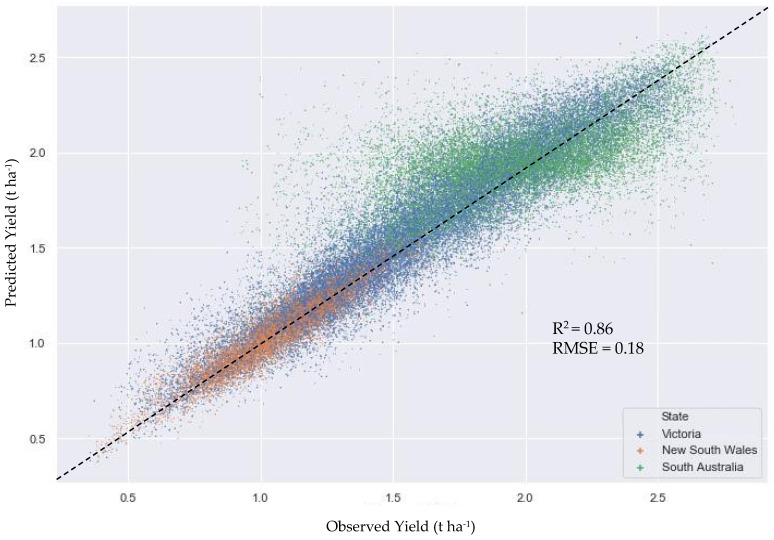
Scatterplot of observed and predicted yield of VIC, NSW and SA paddocks combined.

As seen in Figure 3, the datapoints were mostly closely clustered around the reference line, particularly for yield values between 0.8 to 1.3 t ha^−1^. However, they were more dispersed between the 1.3 to 2.8 t ha^−1^ yield. While the VIC paddock (blue) yield values were broadly distributed, NSW paddock (orange) yield values tended towards the lower, and for SA paddock (green), the higher ranges. 

**Figure 4:**
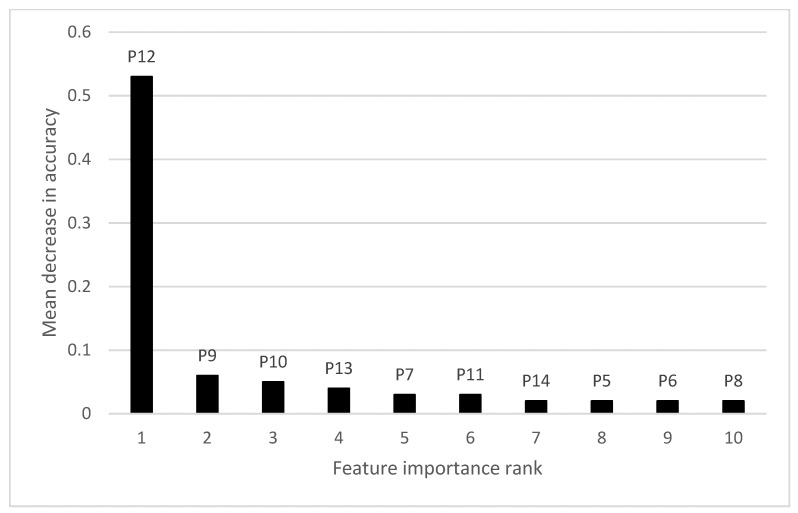
Top 10 features of importance for regional RF yield prediction model. Note: Data labels e.g., P12 refer to NDVI in Periods described in Table 2.

Feature importance analysis found that NDVI data acquired in late September/early October were most important to the prediction accuracy of the RF model developed for the 3-paddock composite (Figure 4; Table 2). This corresponded to 142, 179 and 145 DAS for VIC, NSW and SA paddocks, respectively. If the NDVI data for Period 12 (P12) were excluded as input to the model, a mean decrease in prediction accuracy of 53% occurred. In contrast, excluding NDVI data from later or earlier time Periods led to only 2% to 6% mean decrease in prediction accuracy. Notably, only NDVI images from P5 to P14 featured in the top 10 most important features.

We also found low feature importance of weather (temperature and rainfall) datasets, being ranked outside of the top 10; this also applied to the individual paddock RF prediction models discussed below.

### 3.2. Individual Paddock Yield Prediction Models

For all three paddocks, predicted mean yields were very close to the observed mean yield with less than 1% difference (Table 5). Standard deviation values showed that RF model predictions resulted in lower variations around the mean compared to observed yield, with the worst performance for SA paddock and best performance for NSW paddock. This was also shown in the overprediction of minimum yields by up to 0.06 t ha−1 for NSW paddock, and underprediction of maximum yields by up to 0.14 t ha^−1^ for SA paddock.

The individual paddock RF model performance metrics are presented in Table 6. RF prediction models for VIC and NSW paddocks performed well with high *R*^2^ values, although with only moderate performance for the SA RF prediction model with *R*^2^ at 0.447. Nevertheless, all adjusted *R*^2^ values indicated the absence of overfitting. *MAE*, *MSE* and *RMSE* values were generally good, with lowest values for the NSW paddock but for the SA paddock, relatively higher error values.

**Figure 5:**
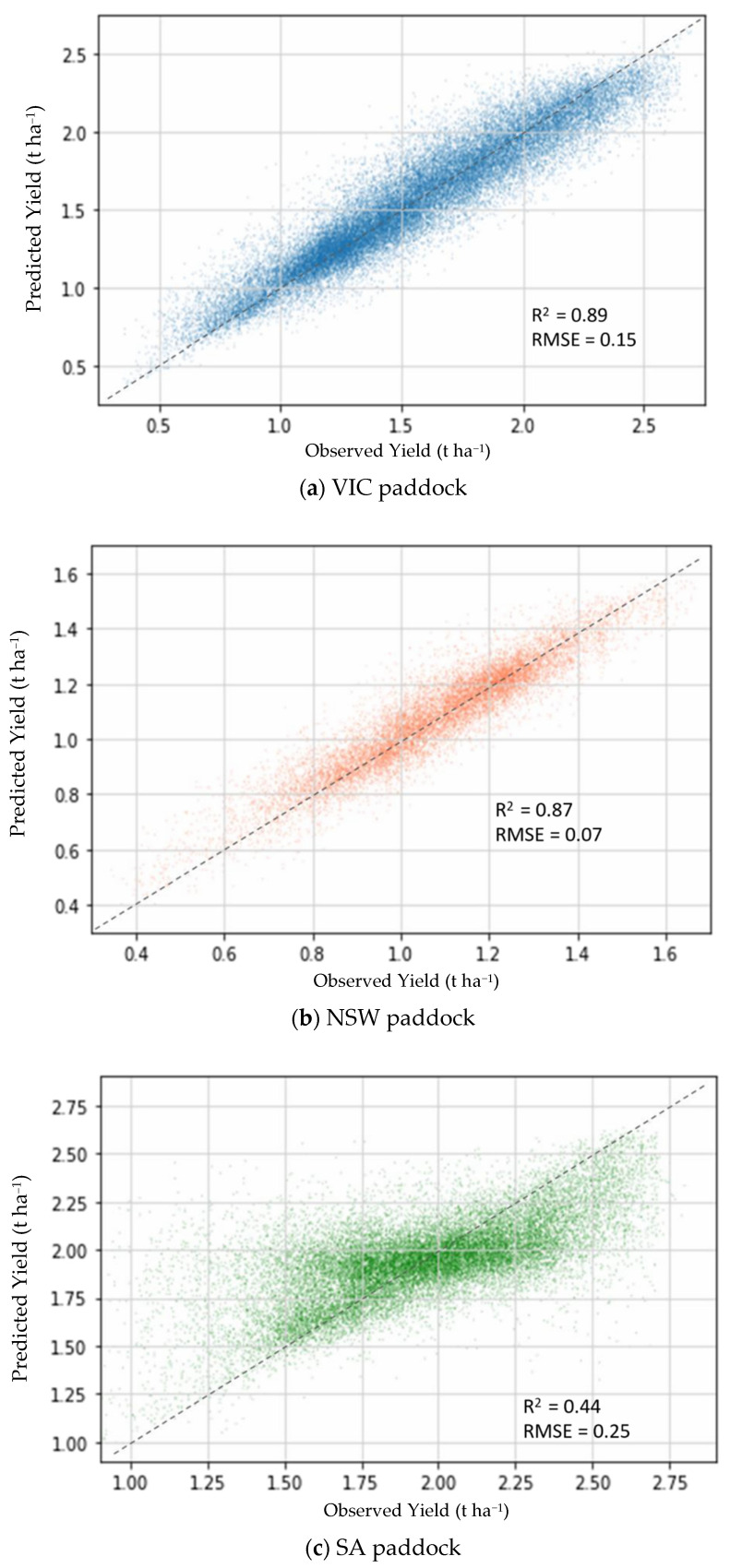
Comparison of predicted vs. observed yield for (**a**) VIC; (**b**) NSW and (**c**) SA paddocks.

Figure 5a–c compare the RF predicted and observed yield for VIC, NSW and SA paddocks, respectively. There was a close clustering of data around the reference line for VIC paddock for yield values between 1.0 to 1.3 t ha^−1^, while this was seen for the NSW paddock between 0.8 to 1.3 t ha^−1^. SA paddock displayed quite widely-dispersed values around the reference line with clear underprediction 2.0 t ha^−1^ and overprediction below it.

**Figure 6:**
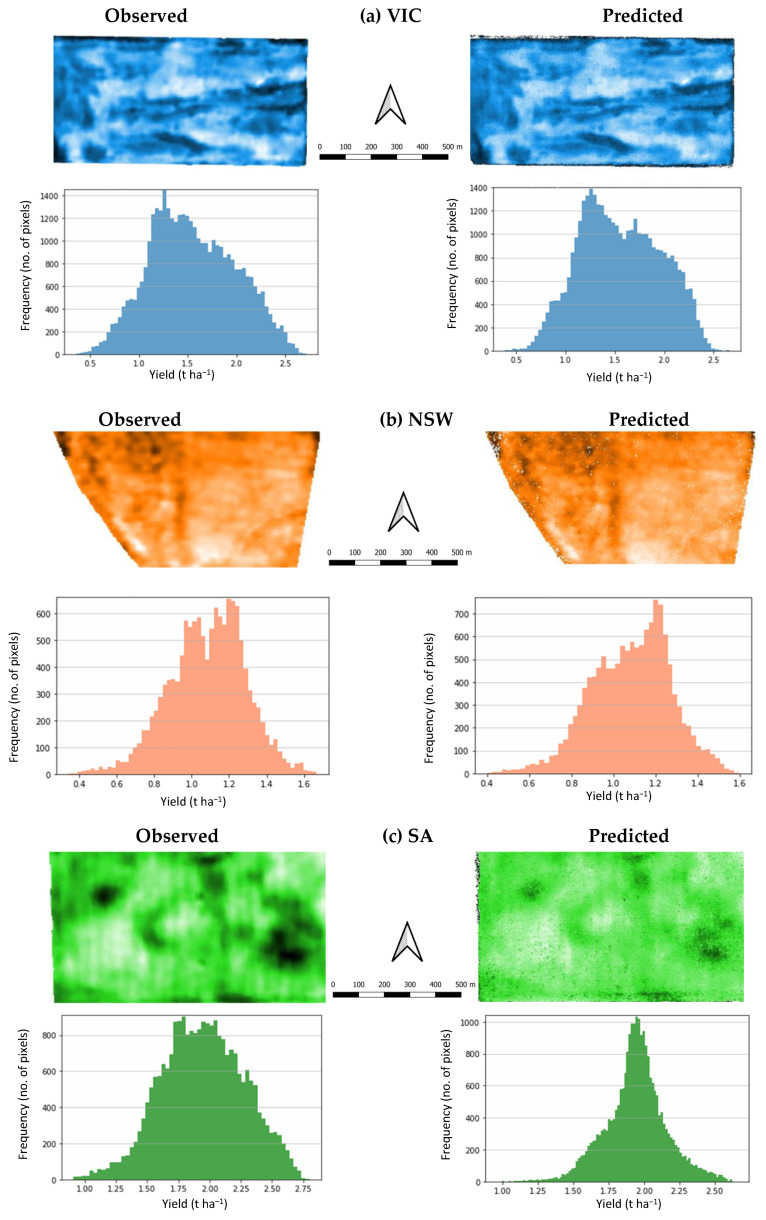
Yield maps and histograms for (**a**) VIC; (**b**) NSW and (**c**) SA paddocks. Notes: Yield maps—darker colors indicate higher yield values; yield histogram y-axes differ in range for NSW and SA paddocks.

Figure 6a–c present the yield map and histogram for VIC, NSW and SA paddocks, respectively. For the VIC paddock, we saw from the yield map, good spatial correspondence between the observed and predicted values. The histograms showed a higher number of high-yield values being predicted compared to the observed yield values, quite apparent for the yield values above 2.0 t ha^−1^. The NSW paddock yield map also showed good spatial correspondence between observed and predicted values. The NSW yield histograms also showed good similarities in the general distribution of values, although the prediction was not able to replicate the bimodal pattern of the observed yield with peaks at 1.0 and 1.3 t ha^−1^. The prediction gave a single high peak around the 1.25 t ha^−1^ yield value. The SA paddock yield map had comparatively poorer spatial correspondence between the observed and predicted values. The predicted yield histogram had a higher peak of average values around 1.95 t ha^−1^ compared to the observed yield histogram, which had gentler peaks around 1.75 t ha^−1^ and 2.15 t ha^−1^. This corroborated with the lower standard deviation of 0.22 t ha^−1^ for predicted yield compared to 0.33 t ha^−1^ for observed yield in Table 5. 

### 3.3. Feature Importance Analysis for Individual Paddocks

Table 7 shows the mean decrease in accuracy (MDA)—the arithmetic averaged loss of prediction accuracy for all individual pixels comparing predicted output with target output values, if one of the features were excluded as predictor input for the RF model, for the top 10 most important features, and the corresponding Period (P) (Table 2) of the NDVI data.

For the VIC paddock, NDVI data for Period 12 (30 September to 15 October; 138 to 153 DAS), with the imagery on 4 October (142 DAS) used for the VIC RF yield prediction model. This image contributed 68% to the prediction accuracy. The second most important NDVI map in the Period 11 (20 September, 128 DAS) contributed 11% to prediction accuracy.

For the NSW paddock, NDVI data for Period 10 (29 August to 13 September; 147 to 162 DAS), with imagery obtained on 4 September (153 DAS) used for the NSW RF yield prediction model. This image contributed 68% to prediction accuracy. 

For the SA paddock, NDVI data for Period 7 (12 July to 27 July; 64 to 79 DAS) with imagery obtained on 14 July (66 DAS) used for the SA RF yield prediction model. In contrast to the results for VIC and NSW paddocks, this image contributed only 22% to prediction accuracy. The second most important NDVI map was obtained in Period 10 on 4 September (118 DAS) contributing 12% to prediction accuracy. Distribution of RF yield prediction model feature importances of NDVI data for SA paddock were hence more evenly distributed across the growing period, albeit with lower prediction accuracy.

## 4. Discussion

The regional-scale RF regression model was able to provide accurate wheat yield prediction at a high *R*^2^ value of 0.86 and low *RMSE* of 0.18 t ha^−1^. The results show that the model is robust at prediction across the three different paddocks with distinct conditions. Despite its limitations, NDVI continues to be a useful Vegetation Index (VI) for yield prediction, and the results from this study concurs with previous work using UAV-mounted cameras [57], LANDSAT [58] and MODIS imagery [59]. The present work further demonstrates the ability for spatially-explicit predictions by using high-resolution imagery and machine learning (RF) approach. Furthermore, the high-cadence of Planet imagery enabled the acquisition of cloud-free images of our target paddocks within a constrained time period, an important consideration for operational applications at the regional and local scales.

Interestingly, we found that the weather data were not significant features for all developed RFR yield prediction models, even though it is indubitable that these are important factors affecting crop health and growth [60], and their inclusion have improved accuracy of various yield prediction techniques [61,62,63]. None of the weather data layers were found in the top ten features of importance. The key explanation could be that while NDVI is able to indicate plant health, including their responses to varying weather and climatic conditions [64], high spatial resolution 3 m NDVI used in this study (and indeed other VIs), the precision with which plant growth conditions are reflected, and the fidelity with which the data can be extrapolated via RFR to reasonably accurate yield predictions, render near-term weather data unnecessary. Hence, RFR could enable parsimonious wheat yield prediction models to be built by possibly precluding the requirement for accessing and assembling large weather datasets to aid the prediction process.

While good agreement was found between predicted and observed yield, the reported differences can be attributed to several factors. Firstly, NDVI estimates live vegetative biomass [65] which has good, but not perfect correspondence with yield. This is especially so for grain crops, such as wheat, where the yield comprises grains in storage organs in contrast with pasture or forage crops. Secondly, temperature extremes such as frost damage to foliage, particularly during winter, can initiate leaf senescence and lower vegetation greenness (higher red reflectance, lower NDVI value) but lag in time for these to manifest (i.e., in later images normally of lower importance). The yield impacts of frost, particularly during critical periods during the reproductive and grain development phases, strongly determine wheat grain number and size [46]. Thirdly, index value saturation, and obscuring of the biomass beneath the closed canopy can lead to high uncertainty in biomass estimates [66,67] and, consequently, wide variation in accuracy of yield predictions. 

Model calibration required few tunable model parameters, similar to how Houborg and McCabe [42] found good accuracy by simply optimizing the number of trees (*n_estimators*). This study concurs with other studies across different crops including wheat (biomass) [28], sugarcane [21] mango [68] and corn [69]. Thus, RFR has been found to be a suitable and parsimonious technique for regional-scale wheat yield prediction.

Examination of RFR yield predictions for individual paddocks found good accuracy for VIC and NSW paddocks. The most important NDVI data for these two paddocks correspond well to the start of anthesis where peak biomass (and NDVI values) are likely to translate predictably to grain yield [70], barring any unpredictable perturbations in the intervening time to harvest, such as temperature stresses (heat/frost) or strong winds causing lodging. These demonstrate the viability of RFR for aspatial paddock-level prediction of mean yields, as well as the good accuracy of spatially-explicit pixel-level yield predictions in the given conditions. However, SA paddock RFR prediction model outputs lowered the overall regional prediction accuracy, and had only moderate accuracy at the individual paddock level illustrated by substantial statistical and spatial differences between the predicted and observed yields. The SA RF model feature importances’ lower values, more even distribution, and higher importance of earlier Periods (Table 7) indicate that some unpredicted factors were not comprehensively accounted for, when compared to the VIC and NSW RF model prediction accuracies and feature importance analyses. Such inter-regional variability could be mainly attributed to the inter-paddock differences in wheat variety, sowing density, soil, topography, local weather conditions and farmer management practices, not all of which can be pragmatically quantified. The earlier time of most important NDVI map in P7 was likely to have coincided with tillering stage [70], but was probably confounded by paddock-level variabilities and perturbations affecting crop health and yield later in the season.

We noted that only SA paddock had legume residue from the previous crop, which has been shown to supply additional N to wheat crop via fixed soil N as well as organic N that mineralized as it decomposed [71] and therefore enhanced yield outcomes. However, there would likely to have been substantial spatial heterogeneity in these decomposition processes [72]. Furthermore, Scepter was one of the highest yielding and drought tolerant varieties (trial mean yield: 3 t ha^−1^) during the 2018 NVT [73]. Hence, this could have led to ample overall biomass growth, canopy closure and NDVI value saturation which could have obfuscated the predicted yield, leading to the overprediction of lower- performing areas and underprediction of high-performing areas. This may not have happened on the VIC and NSW paddocks which grew comparatively lower-yielding and less drought-tolerant varieties. This uncertainty would have been compounded by the variable topography and soil characteristics for the SA paddock. For instance, we observed high-frequency microtopographical variations over SA paddock compared to the more regular undulating terrain for VIC and flat terrain for NSW paddocks. For SA paddock, the high intra-paddock soil variability, and corresponding soil moisture and fertility variations, could have contributed to substantial uncertainties in yield outcomes. This corresponds to how study [74] found close relationships between yield and mean surface curvature due to correlations with soil productivity (e.g., moisture).

Furthermore, haying-off [75], leading to reduced yields due to post-anthesis drought and heat stress despite vigorous growth through the season (detected as high NDVI values) aided by ample N supply, can be quite unpredictable at both the regional and paddock-level scale. This could have contributed to the overprediction of yield, particularly in SA paddock. For instance, SA paddock recorded maximum daily temperatures above 35 °C for three consecutive Periods prior to harvest, compared to 1 each for VIC and NSW. These numerically small occurrences may not have been adequately accounted for amongst all the other feature datasets used in the RFR. Altogether, the high spatio-temporal variability in crop phenology throughout the season with drought and heat stresses led to only moderate prediction accuracy for the SA paddock at Pinery. These results highlight nuances in crop phenology, and their variable presentation via satellite imagery and NDVI that are difficult to capture even using RFR. 

The results for the composite dataset, and exemplified by the SA paddock results, show that poor prediction accuracies occurred at the lower and higher ends of the yield values. Similar outcomes were also found by [27] who found that while overall accuracy of RF yield predictions were excellent, poor accuracy was found at extreme values or for values that were outside the range of the training dataset. Nevertheless, similar to study [59], the developed RF yield prediction models were able to predict yields up to two months before harvest, a timeline that is useful for farmers and other wheat crop stakeholders further along the value chain. 

The results of the present study for wheat yield prediction (regional RF model: *R*^2^ = 0.86, *RMSE* = 0.18 t ha^−1^, n = 75,495) compare favorably with similar studies such as [28], who reported for wheat biomass prediction using RF and HJ-1/2 30 m satellite imagery, *R*^2^ = 0.79, *RMSE* = 1.81 t ha^−1^ (n = 49); [76] applied RF yield prediction methods to wheat, barley and canola using MODIS 250 m derived Enhanced Vegetation Index and reported Lin’s Concordance Correlation Coefficient (LCCC) of 0.89 to 0.92 at the field resolution (*RMSE* = 0.36 to 0.42 t ha^−1^) at 10 m spatial resolution. Relatedly, study [30] evaluated convolutional neural networks (CNN) with bootstrapped regression trees (BRR), and the effects of different data quality and resolution (Landsat 8, Sentinel 2 and proximal sensing) at 5 m spatial resolution; they found optimal wheat yield predictions at LCCC = 0.63 (*RMSE* = 0.08) for three selected fields using BRR with Sentinel 2 data. The results from this study, particularly at the paddock scale for VIC and NSW paddocks at 3 m spatial resolution, demonstrate the viability of RF modeling and, more broadly, data and ML-driven techniques for wheat prediction. The spatial variations in predicted as well as observed yield are particularly helpful in the era of precision agriculture where farmers are able to make better spatial accuracy scouting or fertilizer management decisions (e.g., via management zoning) [76]. 

Key challenges involved in this work include the inability to evaluate various other Vegetation Indices (VIs) that could enable even higher prediction accuracies. Although the broad spectral resolution limited the range and precision of vegetation indices that could be harnessed, this study showed that good yield prediction results were possible by using RF algorithms with NDVI data. This also points to the high potential for further work using other VIs such as chlorophyll content index (CCCI) or Photochemical Reflectance Index (PRI) [77], as air and spaceborne platforms with more spectral bands become available as sensor technologies advance [78].

RF algorithms have some limitations which the present research encountered and researchers should be aware of. Dang et al. [79] highlighted that the lower performance of RFR autumn crop yield prediction compared to Support Vector Regression (SVR) and Deep Neural Network (DNN). This was attributed to its inability to make predictions beyond the range of values of the training set data, the tendency of overfitting when modeling noisy data, and discreteness of output values defined by categories (however narrowly defined), which would otherwise give continuous range of output values provided by, e.g., SVR. 

This research also did not integrate data reflecting field management practices such as fertilization and pest management. Although these are important factors affecting crop health and yield [80], it is typically very difficult to obtain such information in a timely way from farmers at the individual level, as well as prepare and input them into the model. It is also likely the effects of these practices manifest in the crop performance and health for which the spatially-distributed NDVI and yield values reflect to a reasonable extent, although with some time lag. Thus, excluding management practices data is not critical to the yield prediction objectives while allowing the RF modeling process to stay as parsimonious as possible.

Beyond the present research, further work can include (i) increasing or decreasing temporal resolution of predictor variables (e.g., NDVI) to optimize modeling and data processing times and higher accuracy; (ii) evaluation of other VIs or the use of different VIs at different growth stages [74]; (iii) increased number of paddocks distributed throughout the region to increase size of training datasets and to capture greater variability for better model generalizability; (iv) evaluating RFR yield prediction models for other areas such as the western Australian wheat belt; (v) comparative evaluation of RFR with other ML algorithms such as SVR, DNN, Least Absolute Shrinkage and Selection Operator (LASSO) and Sequential Forward Selection (SFS) [81]. At the time of writing in late 2021, southeast Australia and much of the rest of the country is estimated to record harvests at least 10% above the 10-year average [77]. Application of the RF modeling method to this “good” growing season in contrast to the “difficult” season examined in this research would help to further test its robustness and viability for operational use, as well as reexamine the importance of various features such as weather parameters, and the integration of spatially-explicit soil data [28].

## 5. Conclusions

This study evaluated the use of RFR to perform in-season wheat yield prediction at regional and paddock-level scales in southeast Australia using (3 m) NDVI data derived from high-cadence, high-resolution (3 m) PlanetScope satellite imagery and weather data through the winter crop-growing season with actual yield data as the reference. Evaluation of the RFR models found that good yield prediction results were possible by using NDVI data, even though the broad spectral resolution limited the range and precision of vegetation indices that could be harnessed. 

With high accuracy at the regional scale and for two out of three paddocks at the paddock scale, this research shows how RFR-driven yield prediction could be successfully performed in data-rich, information-poor (lack of information on soil, topography, farmer management actions) contexts. Hence, RFR methods have much potential for regional-scale surveillance and monitoring of wheat crop that can benefit various business stakeholders, while paddock-level yield predictions can aid spatially-explicit tactical crop management, harvest and post-harvest decision-making by farmers. When fully or partially automated, the modeling outputs can be generated efficiently, accurately and communicated effectively to various stakeholders for timely decision-making. Where yields with significant departures from the mean in terms of amount (t ha^−1^) or quality (protein, grain size), further investigations of the contributing factors (soil, pests, microclimate) can be done. Additionally, the high spatio-temporal resolution of Planet CubeSatCubeSat data exploited by RFR modeling can also be particularly relevant in smallholder farm contexts (e.g., economically less-developed countries) where plot sizes are modest compared to industrial- scale paddocks in countries such as Australia.

## Figures and Tables

**Table 1:** Location, cropping, climate and soil characteristics of study paddocks.

Location	2018 Wheat Crop Information	Paddock Area & Cropping Sequence 2015–2016–2017	Climate	Soil Description
Ouyen, VIC 142.37 E 35.12 S	Variety: *Kord* Sowing: 15 May Harvest: 30 Nov Growing days: 199 Mean yield: 1.53 t ha^−1^	181.2 ha Barley–Wheat–*Fallow*	Mean Max Temp: 23.8 °C Mean Min Temp: 9.8 °C Mean Annual Rainfall: 331.2 mm	Calcarosol (dune systems with series of alkaline sandy/loamy duplex, and sandy clay soils).
Barmedman, NSW 147.46 E 34.15 S	Variety: *Lancer* Sowing: 4 April Harvest: 27 Jan Growing days: 298 Mean yield: 1.06 t ha^−1^	67.6 ha Canola–Wheat–Canola	Mean Max Temp: 24.0 °C Mean Min Temp: 9.9 °C Mean Annual Rainfall: 470.9 mm	Brown Vertosol (heavy clay soil, alkaline with strongly sodic subsoil).
Pinery, SA 138.46 E 34.32 S	Variety: *Scepter* Sowing: 9 May Harvest: 11 Dec Growing days: 216 Mean yield: 1.95 t ha^−1^	120.1 ha Wheat–Wheat–Lentils	Mean Max Temp: 23.6 °C Mean Min Temp: 9.7 °C Mean Annual Rainfall: 408.9 mm	Calcarosol (alkaline silty clay loam to medium-heavy clay) variable soil profiles on dune systems.

**Table 2:** PlanetScope imagery fortnightly Periods, dates and corresponding Days After Sowing (DAS) in year 2018 for each location in the states of Victoria (VIC), New South Wales (NSW) and South Australia (SA), Australia.

Location	Ouyen, VIC		Barmedman, NSW		Pinery, SA	
Period	2018 Date	DAS	2018 Date	DAS	2018 Date	DAS
**1**	-	-	19 April	15	-	-
**2**	-	-	30 April	26	-	-
**3**	25 May	10	14 May	40	16 May	7
**4**	31 May	16	29 May	55	31 May	22
**5**	14 June	30	22 June	79	13 June	35
**6**	30 June	46	30 June	87	29 June	51
**7**	14 July	60	14 July	101	14 July	66
**8**	29 July	75	12 August	130	29 July	81
**9**	13 August	90	27 August	145	26 August	109
**10**	7 September	115	4 September	153	4 September	118
**11**	20 September	128	21 September	170	17 September	131
**12**	4 October	142	30 September	179	1 October	145
**13**	19 October	157	18 October	197	19 October	163
**14**	4 November	173	11 November	221	2 November	177
**15**	18 November	187	26 November	236	17 November	192
**16**	-	-	12 December	252	-	-

**Table 3:** Descriptive statistics for regional-scale observed and RF model predicted yield.

	Observed Yield	Predicted Yield
sample size, *n*	75,495	75,495
minimum (t ha^−1^)	0.35	0.38
maximum (t ha^−1^)	2.79	2.67
mean (t ha^−1^)	1.60	1.60
standard deviation (t ha^−1^)	0.47	0.44

**Table 4:** Statistical performance of regional-scale RF yield prediction model.

Metric	Test Dataset	Validation Dataset
R Squared (*R*^2^)	0.858	0.860
Adjusted R Squared (*R*^2^)	0.858	0.860
Mean Absolute Error (*MAE*)	0.126	0.126
Mean Squared Error (*MSE*)	0.032	0.031
Root Mean Squared Error (*RMSE*) (t ha^−1^)	0.179	0.177

**Table 5:** Descriptive statistics for predicted yields from individual RF models compared with observed yields for VIC, NSW and SA paddocks.

	VIC (n = 37,773)		NSW (n = 13,566)		SA (n = 24,156)	
Yield Statistic (t ha^−1^)	Observed	Predicted	Observed	Predicted	Observed	Predicted
mean	1.55	1.56	1.08	1.08	1.95	1.94
standard deviation	0.44	0.41	0.20	0.19	0.33	0.22
minimum	0.36	0.38	0.34	0.40	0.91	0.96
maximum	2.72	2.66	1.67	1.59	2.80	2.66

**Table 6:** Statistical performance of VIC, NSW and SA RF yield prediction models.

	VIC		NSW		SA	
Metric	Test Dataset	Validation Dataset	Test Dataset	Validation Dataset	Test Dataset	Validation Dataset
*R* ^2^	0.890	0.887	0.870	0.878	0.447	0.443
Adjusted *R*^2^	0.890	0.887	0.869	0.877	0.445	0.441
Mean Absolute Error (*MAE*)	0.110	0.111	0.056	0.054	0.186	0.185
Mean Squared Error (*MSE*)	0.021	0.022	0.005	0.005	0.061	0.060
Root Mean Squared Error (*RMSE*) (t ha^−1^)	0.146	0.147	0.073	0.071	0.246	0.246

**Table 7:** Top ten most important features for VIC, NSW and SA paddock RF models, and corresponding NDVI Period and mean decrease in accuracy (MDA) if excluded.

Feature Importance Rank	VIC		NSW		SA	
	NDVI Period	MDA	NDVI Period	MDA	NDVI Period	MDA
1	18	0.68	16	0.68	13	0.22
2	17	0.11	17	0.14	16	0.12
3	20	0.04	7	0.02	18	0.09
4	13	0.03	21	0.02	15	0.08
5	12	0.03	18	0.02	14	0.07
6	16	0.02	19	0.02	17	0.06
7	15	0.02	20	0.02	12	0.06
8	19	0.02	13	0.02	22	0.06
9	14	0.02	14	0.01	19	0.04
10	11	0.01	9	0.01	11	0.04

## Data Availability

Data is currently withheld due to farmer privacy concerns.
